# Supplementary material for: The interactome of intact mitochondria by cross-linking mass spectrometry provides evidence for coexisting respiratory supercomplexes
Source: Mol Cell Proteomics. 2017 Dec 8;17(2):216–32. doi: 10.1074/mcp.RA117.000470 (PMC5795388; doi:10.1074/mcp.RA117.000470)
Supplement: Supplemental Data [file supp_RA117.000470_133922_0_supp_23978_fzffwf.zip › spectra_annotation/mito_spectra_annotation/60-1-1-1-2-1.pdf]

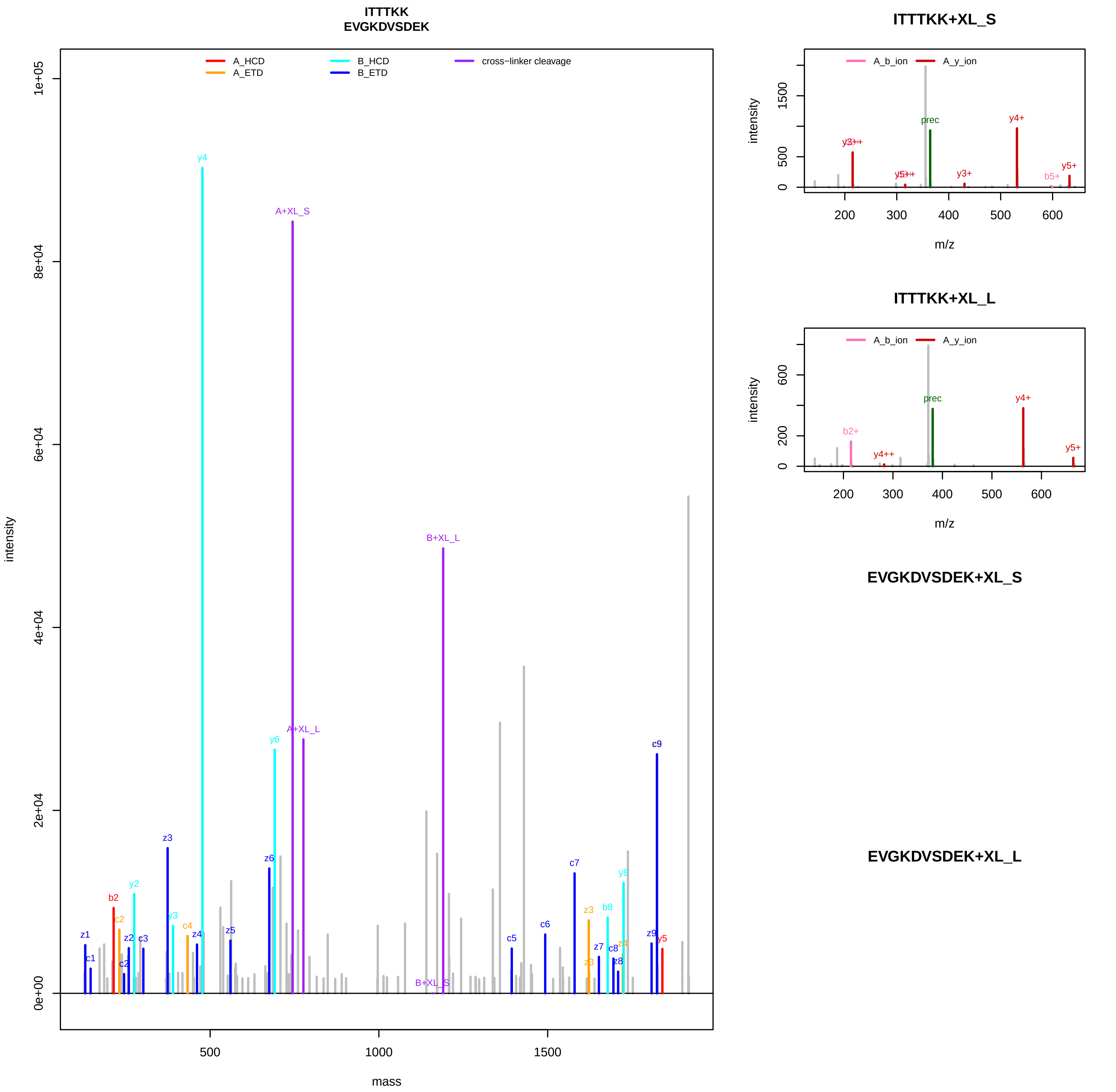

ITTTKK
EVGKDVSDEK
A_HCD
A_ETD
B_HCD
B_ETD
cross−linker cleavage
y4
A+XL_S
B+XL_L
A+XL_L
y6
c9
z3
z6
c7
y8
b8
z3
c6
c5
z1
c1
b2
c2
y2
z2
c3
y3
c4
z4
z5
B+XL_S
z7
c8
z8
z9
y5
intensity
mass
0e+00
2e+04
4e+04
6e+04
8e+04
1e+05
500
1000
1500
ITTTKK+XL_S
A_b_ion
A_y_ion
prec
y4+
y3++
y5++
y3+
b5+
y5+
intensity
m/z
ITTTKK+XL_L
A_b_ion
A_y_ion
prec
y4+
b2+
y4++
y5+
intensity
m/z
EVGKDVSDEK+XL_S
EVGKDVSDEK+XL_L
